# CRISPR-mediated targeted mRNA degradation in the archaeon *Sulfolobus solfataricus*

**DOI:** 10.1093/nar/gku161

**Published:** 2014-03-06

**Authors:** Ziga Zebec, Andrea Manica, Jing Zhang, Malcolm F. White, Christa Schleper

**Affiliations:** ^1^Department of Ecogenomics and Systems Biology, University of Vienna, Archaea Biology and Ecogenomics Division, Althanstr. 14, 1090 Vienna, Austria and ^2^Biomedical Sciences Research Complex, School of Biology St Andrews University, St Andrews KY16 9ST, UK

## Abstract

The recently discovered clustered regularly interspaced short palindromic repeat (CRISPR)-mediated virus defense represents an adaptive immune system in many bacteria and archaea. Small CRISPR RNAs cause cleavage of complementary invading nucleic acids in conjunction with an associated protein or a protein complex. Here, we show CRISPR-mediated cleavage of mRNA from an invading virus in the hyperthermophilic archaeon *Sulfolobus solfataricus*. More than 40% of the targeted mRNA could be cleaved, as demonstrated by quantitative polymerase chain reaction. Cleavage of the mRNA was visualized by northern analyses and cleavage sites were mapped. *In vitro,* the same substrates were cleaved by the purified CRISPR-associated CMR complex from *Sulfolobus solfataricus*. The *in vivo* system was also re-programmed to knock down mRNA of a selected chromosomal gene (β-galactosidase) using an artificial miniCRISPR locus. With a single complementary spacer, ∼50% reduction of the targeted mRNA and of corresponding intracellular protein activity was achieved. Our results demonstrate *in vivo* cleavage of mRNA in a prokaryote mediated by small RNAs (i.e. analogous to RNA interference in eukaryotes) and the re-programming of the system to silence specific genes of interest.

## INTRODUCTION

RNA interference represents one of the many sophisticated mechanisms of gene regulation in eukaryotes and can also act against invading viruses and other genetic elements (1). Recently, an adaptive immune system has been described in bacteria and archaea that also acts through small RNAs. These clustered regularly interspaced short palindromic repeat RNAs (crRNAs) are encoded in CRISPR regions in the genomes that harbor short but mostly unique DNA sequences (spacers) separated by repeats (2). On transcription, the crRNAs are cleaved from the long primary transcript, so-called precursor CRISPR RNA (pre-crRNA), in the repeat sequences and form ribonucleoprotein complexes with CRISPR-associated (Cas) protein(s) (3–7). These are able to target and cleave invading DNA molecules that exhibit complementarity to the guide crRNA (8–10). Acquisition of additional spacer sequences from newly invading viruses or plasmids allows adaptation of the immune system and inheritance to progeny cells (8,11–14). A huge variety of the CRISPR-Cas systems (2) has been found in ∼50% of all bacterial genomes and ∼90% of the archaeal genomes (15). *In vivo* activity has been demonstrated in six model systems of bacteria and in three model organisms of archaea, proving the effective CRISPR-mediated immunity against DNA of invading viruses or plasmids by type I, type II and type IIIA CRISPR systems (5,8,16–22). Furthermore, the type II system (17) has recently been used to efficiently recognize and cleave specific genes in a programmable dual-RNA manner (9) that functions independently and can be used in various bacteria and eukaryotes to generate targeted mutations (23–26). The same system was also engineered to repress gene expression in a bacterium by directing a protein to the promoter to physically block transcription initiation (26,27). Interestingly, some evidence has accumulated that CRISPR-Cas systems can not only target DNA but also RNA molecules. *In vitro* studies in the two hyperthermophilic archaea *Pyrococcus furiosus* (28,29) and *Sulfolobus solfataricus* (30) have demonstrated that crRNAs in association with the so-called CMR protein complex encoded in a type IIIB CRISPR-Cas module specifically lead to cleavage of RNA targets but not DNA. More recently, a type II CRISPR-Cas module was shown to mediate bacterial virulence and immune invasion by affecting mRNA expression (31), but whether mRNA was degraded or transcription was physically blocked was not investigated.

We and others have recently established an *in vivo* study system for the hyperthermophilic archaeon *S. solfataricus* that allowed characterizing CRISPR-mediated DNA interference of plasmid and virus DNA (18,19). Strains of *S. solfataricus* possess a rather complex and extended CRISPR-Cas assembly with five Cas modules classified into the CRISPR-Cas system types I, type IIIA and IIIB (2,32). With the help of our shuttle-vector system that is based on the lysogenic virus SSV1, we have recently demonstrated that DNA interference can be abolished when there is complementarity between the repeat-derived 5′-handle of the crRNA and the protospacer adjacent sequence (PAS) on the virus (33). This mechanism that obviously protects the host from degradation of its own genome was earlier shown in the bacterium *Staphylococcus epidermidis* (16). Even complementarity of only three nucleotides between handle and PAS enabled self-recognition in *Sulfolobus*, i.e. abolished DNA interference (33). In this study, we use this knowledge to shut-off DNA interference in *S. solfataricus* and demonstrate CRISPR-mediated cleavage of specific mRNAs *in vivo*. Cleaved mRNA products are visualized and *in vivo* cleavage sites are mapped. We also re-program the RNA interference system such that it can be used to specifically target the mRNA of chromosomal genes.

## MATERIALS AND METHODS

### *Sulfolobus* cultures and transfection procedure

Cultures of *S. solfataricus* M18 [*pyr*EF mutant of *S. solfataricus* P1 (34)] were grown at 78°C and pH 3 in basic Brock media (35) and supplemented with enzymatically hydrolyzed casein, i.e. tryptone (BD Biosciences) 0.1% (w/v) and (+) d-Sucrose at 0.2% (w/v). For growth of the uracil auxotrophic mutant M18, uracil at a final concentration of 0.0125 mg/ml was added to the media. Electro-competent cells were prepared as previously described (36) and transfected by electroporation using the following conditions: 1250 V, 1000 Ω, 25 mF and 1-mm cuvettes (37,38). Cultures transfected with D63-7U, D63-HA and A53* were mixed with untransfected M18 cells in Brock salts containing 0.4% gellan gum (Gelrite, Kelco Biopolymers) for plaque assay on Brock media-containing plates (19). Between 20 and 150 plaques were obtained per biological replicate (each having three technical replicates). Therefore, plaque-forming efficiency is expressed as % of positive control (A53*).

After transfection, cells were grown in selective media (without uracil) to OD (600 nm) = 1. Cells were then inoculated into tryptone (BD Biosciences) 0.1% (w/v) media with a reduced sucrose concentration (0.04%) until OD (600) = 0.2–0.3. Expression of the protospacer constructs D63-7U, D63-HA and A53* (control) was induced through the addition of 20% (m/v) arabinose (0.16% final concentration). After 4 h, samples of the cultures were taken for DNA and RNA extraction. In each experiment, at least three biological replicates were sampled and analyzed.

### Nucleic acid preparations

DNA extractions from *S. solfataricus* cells were performed with a basic phenol-chloroform extraction with TENS and TENST (39) as lysis buffers. The entire extracted DNA was treated with RNase (Omega, bio-tek) before further analysis. Isolation of total RNA was done with the *mir*Vana™ kit (Ambion®), followed by DNAseI treatment (Promega™), following manufacturer’s instructions. RNA quality and purity were measured by NanoDrop (ND-1000, PeqLab) and agarose gel electrophoresis, respectively. Absence of DNA contamination in the RNA preparation was verified by polymerase chain reaction (PCR) amplification of 100 ng of DNAse I-treated RNA before further analysis. The cDNA reaction was carried out using 1 µg of RNA and M-MuLV Reverse Transcriptase (New England, Bio Labs®) following the manufacturer’s instructions. The cDNA was then purified using columns (NucleoSpin®, Macherey-Nagel) and was used after dilution to ∼ 20 ng/µl for real-time PCR quantification.

### Construction of recombinant viruses

#### General cloning techniques

For RNAi constructs, a protospacer sequence identical to the D63 crRNA (located in the CRISPR locus D of *S. solfataricus* strain P2 and P1) was placed into ORF 406 of the conjugative plasmid pNOB8 (19). The pNOB8 ORF406 was cloned into the gateway entry vector pCR8-GW (Supplementary Figure S3) following the manufacturer’s recommendations (Invitrogen™, TOPO-TA cloning), yielding pEntryA53* (with match to spacer A53 in strain P2 but no match in strain P1). The recombinant plasmid was further mutated using the Overlapping Extension-PCR (OE PCR) method [see following text and (40)] to replace the protospacer A53* by new protospacer sequences matching the crRNA of spacer D63. To maintain the open reading frame of ORF 406, two additional nucleotides were added 5′-flanking to the protospacer (TC, marked in yellow in Supplementary Figure S3). The resulting plasmids (pEntry D63-7U and pEntryD63-HA) were verified by sequencing. Subsequently, they were used in a gateway recombination reaction with the destination vector pDEST-MJ-ara (Supplementary Figure S4A, consisting of the gateway recombination sites, an arabinose-inducible promoter (41) and a terminator region (42) implemented into the *S. solfataricus–Escherichia coli* shuttle vector pMJ03), yielding vectors pMZ-D63-7U, pMZ-D63-HA and pMZ-A53*. For the miniCRISPR constructs, a 900-bp region of CRISPR D locus was amplified from the *S. solfataricus* P2 genome including 497 bp of the leader sequence of the CRISPR locus, six repeats and six spacers (D2 was replaced by a nonsense spacer in the miniCRISPR control). After cloning into the pCR8-GW vector yielding pEntry NBG, OE-PCR was used to replace three spacers (D2, D3 and D4) by a spacer matching the β-galactosidase mRNA at position D2 (BG-HA) (yielding vector pI2). Both miniCRISPR constructs (entry vectors pI2 with spacer BG and pEntryNBG without the BG spacer) were recombined with the destination vector pDEST-MJ (without ara promoter). A scheme of the destination vectors is shown in Supplementary Figure S4.

#### Construct design: OE PCR

OE PCRs were applied for construction of the different virus variants. The OE fragments for D63-U7 and D63-HA were produced by using four partly complementary primers, carrying the desired protospacer sequences flanked by ∼50 nt homologous to the plasmid sequence on both sites of insertion. To create the OE fragment, the overlapping primers were fused by PCR, using a proofreading polymerase (Phusion DNA polymerase®, Finnzyme). For construction of the miniCRISPR construct pI2, six primers were used in the fusion reaction. In the first two PCRs, the OE primers (MOE-FW and MOE-RW) were fused with the spacer-specific primers, yielding two short PCR fragments. In the successive PCR, the two fragments were then fused together and amplified using the primers M-FW and M-RW (Supplementary Tables S2 and S3). Owing to thermodynamic reasons, the OE primers MOE-FW and MOE-RW were not designed on proximal spacers. For this reason, the miniCRISPR-BG-HA lacks spacer D2, D3 and D4 compared with the control but it retains spacer D1, D5 and D6. The OE fragments were used in a ratio of 200:1 as primers in a PCR reaction using 30 ng of the entry vectors pEntryA53* and pEntryNBG, as a template. After amplification, the PCR reaction was digested with *Dpn*I (Fermentas™) to cleave the non-mutated circular plasmid. The digested PCR reaction was directly used to transform *E. coli* (TOP10, Invitrogen™). All recombinant plasmids were verified by standard Sanger sequencing.

### Quantitative PCR

**Figure 1.**
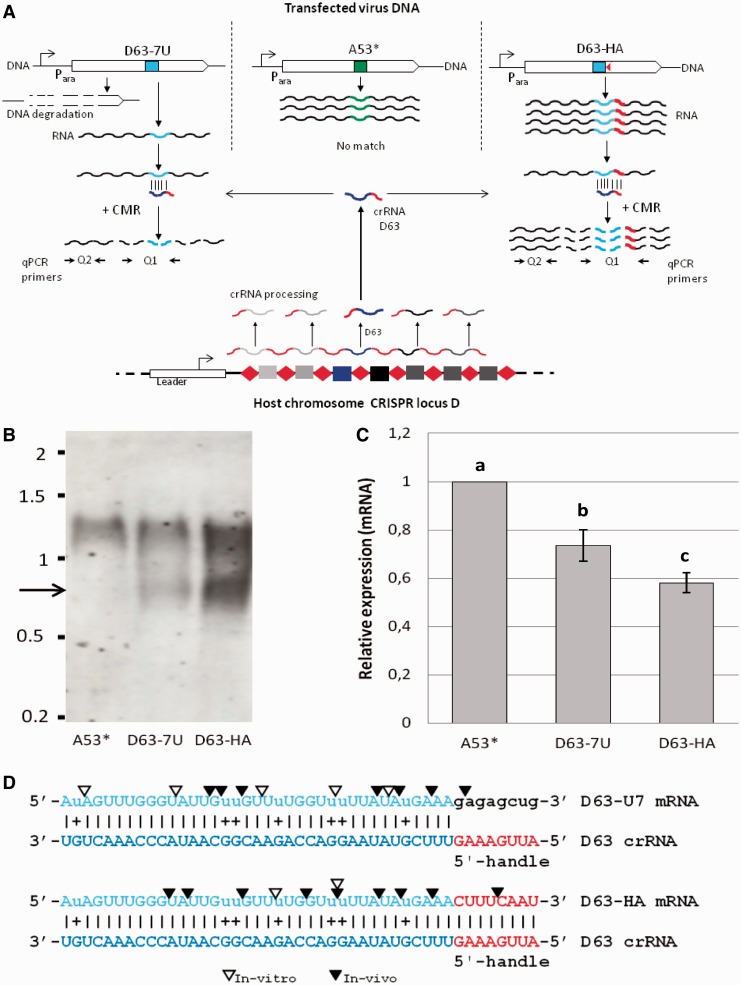
CRISPR-mediated RNA interference of a non-essential gene introduced into *S. solfataricus* P1 via a recombinant virus. (**A**) Schematic representation of virus constructs (top) and chromosomal CRISPR locus with cognate spacer (bottom), as well as respective transcripts (wiggly lines) and their degradation (interrupted lines) A53* = negative control with non-matching protospacer, D63-7 U = seven U:G pairs in RNA:RNA protospacer/spacer hybrid (light blue/dark blue), D63-HA = additional 8-nt 5′-handle matching the repeat-derived crRNA part. P_ara_ = arabinose-inducible promoter. (**B**) Northern blot hybridization showing mRNA cleavage from cells transfected with A53*, D63-7U and D63-HA. Arrow indicates cleavage product. Upper band corresponds to full-length mRNA. (**C**) Results of qPCR showing amounts of mRNA (with primer set Q1 covering the protospacer region; Figure 1A and Supplementary Table S3) over total mRNA (primer set Q2) after reverse transcription of total RNA into cDNA. Lowercase letters indicate significant differences in relative mRNA expression (two-tailed *t*-test, *n* ≥ 3, *P* ≤ 0.028). Error bars, *SD*; (*n* ≥ 3). (**D**) Protospacer/spacer pairs as in (A), capital letters mark complementary bases, lowercase letters mark mismatches, triangles mark cut sites as mapped *in vivo* by RACE (black, Supplementary Figure S1) and *in vitro* by incubating both RNAs with the CMR complex of *S. solfataricus* P2 (white triangles, Supplementary Figure S2A) (30).

**Figure 2.**
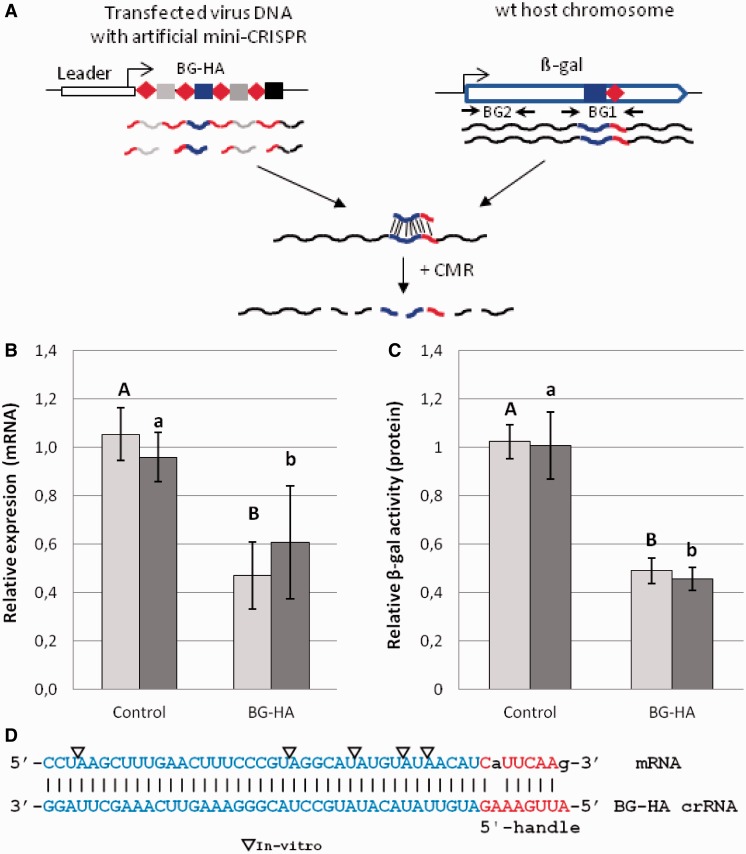
Programmed CRISPR-mediated RNA interference of a chromosomal gene from *S. solfataricus* using a recombinant virus carrying an artificial miniCRISPR locus. (**A**) Schematic representation of miniCRISPR construct with spacer BG-HA targeting the β-Gal gene (SSO3019) in nucleotide positions 679–717, with eight flanking nucleotides exhibiting complementarity to the 8-nt 5′-handle. Locations of primers used in qPCR (BG1 and BG2) are indicated (**B**) Quantitative PCR estimating the relative amounts of mRNA (with primer set BG-Q1 covering the protospacer region) over total mRNA (primer set BG-Q2) after reverse transcription of total RNA. Significant differences in relative mRNA expression between miniCRISPR-BG-HA and control in tryptone and tryptone/arabinose media are represented in capital letters or lowercase letters, respectively (two-tailed *t*-test, *n* ≥ 3, *P* ≤ 0.004). Error bars, *SD* (*n* ≥ 3). (**C**) Significant differences in relative β-gal enzyme activity between miniCRISPR-BG-HA and control in both media are represented in capital letters or lowercase letters, respectively (two-tailed *t*-test, *n* ≥ 3, *P *< 0.00001). Error bars, *SD*; (*n* ≥ 3). (D) Protospacer region in mRNA and 5′-handle and its complement (crRNA). Capital letters: complementarity, lowercase letters: mismatches, triangles: cut sites mapped *in vitro* by incubating both RNAs with the CMR complex of *S. solfataricus* P2 plus ATP (Supplementary Figure S2B) (30).

Each real-time quantitative polymerase chain reaction (qPCR) was performed in triplicate with a SYBR-Green mix (Qiagen™) on an Eppendorf Mastercycler ep gradient S realplex2 (Eppendorf). Two primer sets were designed and used for the qPCR analyses of D63 constructs and in the miniCRISPR experiment for quantification of β-galactosidase mRNA. Primer set Q1 amplified the region of the target site (protospacer) in the ORF406 and β-galactosidase mRNA (i.e. measuring the amount of mRNA that remained uncut), and set Q2 amplified a region located toward the 5′ end not covering the target site (i.e. approximating the totally expressed mRNA level). This approach was used because it avoided any effects of varying virus copy numbers on the gene expression measurements that would have confused results if another mRNA was quantified in the control. Primers and product sizes are given in Supplementary Tables S2 and S3. Plasmids pEntryA53* and pMJ03-05 were used as qPCR standards, and amplification PCR efficiencies ranged between 94 and 99% on the template ORF406 and between 92 and 102% for the β-galactosidase gene. Three biological replicates with three technical replicates each were performed for each experiment. The relative quantification on ORF406 transcripts (D63-7U and D63-HA) was calculated using Q1 products (covering the protospacer, region of cleavage) over total mRNA obtained by Q2 primers compared with the control A53*, using qPCR normalization methods separately for each replica (43). The relative quantification of the miniCRISPR-BG-HA constructs was performed using two control samples: miniCRISPR-control and pMZ-A53* (two tailed *t*-test shows no significant differences between miniCRISPR-control and pMZ-A53*, with *P* = 0.2199 and *P* = 0.2698 for tryptone and tryptone/arabinose, respectively). The relative quantification was calculated by the same principle as mentioned earlier using the primer pairs BG-Q1 and BG-Q2. Significance values, average and *SD* for ORF406 and β-galactosidase are shown in Figures 1C and 2B, respectively.

### Rapid amplification of cDNA ends

Extracted total RNA from the RNAi constructs 406-7U and 406-HA was treated with DNAseI (Promega™), and endpoint PCR was performed to determine absence/presence of remaining DNA. About 1 µg of the treated RNA was polyadenylated with an *E. coli* Poly(A) polymerase (New England, Bio Labs™). The polyadenylated RNA was used in a reverse transcription reaction (M-MuLV Reverse Transcriptase, New England, Bio Labs™) with a poly(T) primer. After retrotranscription, the cDNA was amplified with primer 406-FW3 and a poly(T) primer (57°C annealing temperature), with Phusion DNA polymerase® (Finnzymes). The PCR product was successively purified by agarose gel electrophoresis (Macherey-Nagel) and cloned into a pJet cloning vector (Fermentas™). In all, 25 different clones from D63-7U and 18 for D63-HA were sequenced by standard Sanger sequencing using the primer Q1-FW.

### Northern blot analysis

Total RNA (2.5–10 µg) was denatured for 10 min at 65°C before separation on a 1% formaldehyde gel (80 V, 6 h). After transfer to nylon membrane by capillary blotting (Membrane Hybond-XL, Amersham), the RNA was cross-linked with UV light. The RNA was visualized with 0.4% methylene blue and ladder position was determined. Hybridization was performed with a digoxigenin-labeled (DIG-labeled) dsDNA probe obtained through a PCR reaction with DIG-labeled dNTPs (Roche). Primers in the PCR reaction were Pro-FW1 and Pro-RW2, amplifying 333 nt, at the 5′-end upstream of the protospacer region, not covering the protospacer (Supplementary Table S3). The hybridization was done overnight at 42°C. Three stringent washing steps were made for 30 min each with 0.2% SSC/0.1% SDS at 55°C, 60°C and 65°C. Probe/mRNA hybrids were detected with DIG antibody (Roche) and chemiluminescence reaction with CSPD solution (Roche). Different amounts of total RNA were loaded for the blot shown in Figure 1B and Supplementary Figure S5 (∼2.5 µg for A53*, 10 µg for D63-7U and 5 µg for D63-HA) to allow visualization of the cleavage product of D63-7U.

### Preparation of crude protein extract

In all, 10 ml (OD600 = 0.3–0.4) of transfected *S. solfataricus* M18 cell culture was harvested before and after supplement of arabinose (as mentioned earlier) by centrifugation at 4000 rpm for 15 min at 4°C. Cells were resuspended in 700 μl of 10 mM Tris-HCl buffer, pH 8, and sonicated (Bandelin SONOPULS HD2070, 3 × 1 min on ice, 40% duty cycle). After sonication, cell debris were removed by centrifugation at 15 000 rpm for 30 min at 4°C. Cell-free extract (supernatant) was collected and stored at −80°C until use. Protein concentrations of the different cell extracts were determined using the Bradford method with serum albumin as standard, all in triplicate.

### β-Galactosidase activity assay

Quantitative β-galactosidase assays were carried out to assess the activity of β-galactosidase in the crude cell extracts. Activity was measured by following the enzymatic hydrolysis of *o*-nitrophenyl-β-D-galactoside to galactose and *o*-nitrophenol, and was quantified spectrophotometrically. In all, 50 μl of cell-free extract was added to 950 µl of *o*-nitrophenyl-β-D-galactoside solution (0.84 mg/ml in 50 mM sodium phosphate buffer, pH 6.5) and incubated at 75°C for 5 min. The release of *o*-nitrophenol was then measured spectrophotometrically at 405 nm with intervals of 30 s, and β-galactosidase activity (mU/mg) of the different cell extracts was determined using the Lambert–Beer law, as described earlier (36). Protein expression ratios were calculated by dividing the β-galactosidase activity (mU/mg) of the sample (miniCRISPR-BG-HA) by the β-galactosidase activity of the control sample (miniCRISPR-control), which was set to one. Three biological replicates were performed. Significance values, average and *SD* are shown in Figure 2C.

### *In vitro* RNA cleavage assay

*In vitro* RNA cleavage assays were carried out as described previously (30). In brief, 500 nM purified SsoCMR complex and 100 nM unlabeled crRNA (D63 crRNA) were mixed in buffer [20 mM Mes^.^HCl pH 6.0, 100 mM glutamate, 10 mM DTT, 10 mM MnCl_2_ and the RNase inhibitor SUPERase.in (Ambion®)] in the presence or absence of 100 µM ATP and pre-incubated at room temperature for 10 min before the addition of 500 nM 5′-32P-end-labeled synthetic target RNA to the reaction mix (10 µl total volume). The reaction was further incubated at 75°C for 10 min, then stopped with EDTA and analyzed by gel electrophoresis and phosphor imaging.

### Oligonucleotides for *in vitro* RNA cleavage assay

RNA oligonucleotides were chemically synthesized (Integrated DNA Technologies). The D63 crRNA guide sequence shown here differs by one base from that used previously (30). The original Zhang *et al.* sequence had an extra A at position 32 that was introduced by error. This difference has no effect on the cleavage activity observed.

The sequences are:

crD63: 5′-AUUGAAAGUUUCGUAUAAGGACCAGAACGGCAAUACCCAAACUGU-3′
D63-7U: 5′-CGAAUCAAUAGUUUGGGUAUUGUUGUUUUGGUUUUUAUAUGAAAGAGAGC-3′
D63-HA: 5′-AUAGUUUGGGUAUUGUUGUUUUGGUUUUUAUAUGAAACUUUCAAUGAG-3′
crBG-HA: 5′-AUUGAAAGAUGUUAUACAUAUGCCUACGGGAAAGUUCAAAGCUUAGG-3′
BG-HA: 5′-CCUAAGCUUUGAACUUUCCCGUAGGCAUAUGUAUAACAUCAUUCAAG-3′

## RESULTS AND DISCUSSION

In our previous work, we have demonstrated that the CRISPR system in *S. solfataricus* can recognize and trigger degradation of a protospacer located on viral DNA. This study also showed that the efficiency of plaque formation decreases with an increasing number of mismatches between spacer and protospacer (19,33). Here, the system was modified to circumvent CRISPR-mediated DNA cleavage to study RNA interference. For this purpose, a protospacer was used as a target on the viral DNA that was complementary to crRNA D63 encoded on locus 6 (or D) (44). This crRNA had been shown earlier to be the most prominent crRNA recruited into the CMR complex that is able to degrade RNA *in vitro* (30). Seven point mutations were introduced into the DNA target of virus D63-7U that led to T:G mismatches in a DNA:RNA hybrid but allowed U:G pairing in the corresponding RNA duplex (Figure 1). In construct D63-HA (=HAndle), we have additionally introduced a stretch of 8 nt at the 3′ end of the protospacer called the PAS. This exhibited complementarity to the 8-nt ‘handle’ of the 5′ end of the crRNA. The 8-nt-long 5′-handle, which is derived from the flanking repeat in the chromosome, has been shown to remain attached to the crRNAs on their maturation from the initially produced pre-crRNA (16,28). As shown earlier for the type IIIA system in *Staphylococcus epidermidis* (16), complementarity between handle and PAS abolished DNA interference also in our system (33).

The gene with the respective protospacer was expressed from the virus under the control of an arabinose-inducible promoter. After transfection of DNA and subsequent plaque assays, only around 62% of plaques were observed for construct D63-7U as compared with the control A53* (Table 1). Apparently, seven G-U mismatches in D63-7U allowed the virus to escape the host’s CRISPR system. This effect was sufficient for the virus, such that it could stably establish as a population in liquid culture under selective conditions. In contrast, construct D63 (20% plaque formation) could not survive as a population in culture, even under selective conditions and was not further characterized (Table 1). But more remarkably, no significant DNA degradation was observed anymore for construct D63-HA, which in addition to the seven mutations in its protospacer sequence matched the 5′-handle of the crRNA (92% plaque formation). The observation confirmed our earlier findings, that CRISPR DNA interference modules in the host are circumvented almost completely by a perfectly matching PAS that is complementary to a part of the repeat in the CRISPR locus and, therefore, also complementary to the 5′-handle of the crRNA (33).

**Table 1.** Plaque-forming units normalized to the control A53*

Construct	Average (in %)	*SD* (in %)
D63-DNA	19.3	13.5
D63-7U	62.1	1.3
D63-HA	91.6	8.0
A53*	100	0

*n* = three biological replicates, with each *n* = three technical replicates, *SD* = standard deviation.

We used qPCR on reverse-transcribed total RNA to analyze the amount of cleaved mRNA with two specific primer pairs. The first PCR product (Q1 in Figure 1) covered the region of the protospacer (that was expected to be cleaved, i.e. the target) and the second one covered an upstream region closer to the transcription start of the same respective gene (the reference template). The latter region was not a direct target of the RNA interference and should thus allow an estimate on the total mRNA level of the same gene. As a negative control, RNA from transformants with a protospacer (A53*) was used that did not have a cognate spacer in the chromosome of strain *S. solfataricus* P1 (but only in the closely related strain *S. solfataricus* P2).

The amount of cleaved over uncleaved mRNA is shown in Figure 1C after normalizing it to the ratio of qPCR products obtained from cDNA of the control (A53*). Approximately 42% of mRNA cleavage was observed with the D63-HA construct and ∼26% with D63-7 U (Figure 1C). We assumed that the lower level of cut mRNA in construct D63-7U was because of the shorter mRNA-crRNA hybrid formation, as the protospacer D63-7U is lacking the 8-nt sequence compared with the construct D63-HA that has a perfectly matching PAS to the crRNA D63, expanding the target from 37 nt to 45 nt. Because there was still significant DNA interference with construct D63-7U, we assumed that the lower level of RNA interference was also caused by the overall lower level of virus DNA in the respective culture. This was confirmed by qPCR studies estimating virus DNA levels in the culture (Supplementary Table S1B).

For confirmation of *in vivo* RNA cleavage mediated by the crRNA D63, northern blot analysis (Figure 1B) and rapid amplification of cDNA ends (RACE) were performed to visualize and map the cleavage sites (Figure 1D and Supplementary Figure S1). Beside the uncut full-length mRNA that was the only band in the northern hybridization detected in the control, an additional smaller band of the size expected for mRNA cut in the protospacer site was detected for D63-7U and D63-HA transfectants, whose abundance was in accordance with that measured by qPCR (Figure 1C and Supplementary Table S1A). The 3′ end of the mRNA (downstream of the protospacer) could not be visualized in northern analyses probably because of fast degradation in the cell. More accurate lengths of the cut mRNA species generated through RNA interference in the transformed cells were determined in RACE experiments. About two-thirds of all cut sites in D63-HA and D63-7U transfectants were mapped inside the protospacer regions of the mRNA or in close vicinity, i.e. 3 nt inside the region of the 8-nt handle region, with an accumulation of mRNA ending at AU and UU sites (Figure 1D and Supplementary Figure S1). Other mRNA ends (10 of 25 for D63-7U and 6 of 18 for D63-HA) were located toward the 5′end of the full mRNA, indicating more progressively degraded products (from 3′ to 5′ direction). No cuts were located by RACE beyond the protospacer toward the 3′ end of the full mRNA, strongly supporting the conclusion that initial cutting of the mRNA occurred inside the protospacer and then progressed toward the 5′ end.

### Cleavage of mRNA *in vitro*

To explore if the sequence-specific RNA cleavage observed for the D63-7U and D63-HA targets could be ascribed to the crRNA-dependent ribonuclease activity of the CMR complex, *in vitro* assays were performed as described earlier (30). The CMR complex in *S. solfataricus* has seven different subunits including the large Cas10 subunit and cuts *in vitro* at U/A sites in a reaction that is dependent on manganese and stimulated by ATP (30). The catalytic site of the complex has not yet been determined. A similar CMR system is found in euryarchaea, including *P. furiosus* (28,29). This version of the complex lacks the Cmr7 subunit found in *S. solfataricus* CMR and appears to cleave RNA using a different mechanism that includes a molecular ruler. By contrast, the CSM complex shares the Cas10 subunit but is known to cleave plasmid DNA substrates *in vivo* (10,16).

Small RNAs of D63-7U and D63-HA were synthesized and radiolabeled and their cleavage was assayed *in vitro* using purified CMR complex of *S. solfataricus*. Both substrates were cleaved by the CMR complex in the presence of the crRNA D63, as expected, but not in its absence (Figure 1D and Supplementary Figure S2A). These data confirm our *in vivo* results and show that the CMR complex does not have an absolute requirement for a mismatch opposite the 5′-handle. Interestingly, *in vitro* cleavage of the D63-HA substrate was not stimulated by ATP, whereas cleavage of the other substrate was, which is presumably due to the effect of complete base pairing between the 5′-handle of the crRNA D63 and the D63-HA target (Figure 1D).

The *in vivo* mapped cutting sites (by RACE, Figure 1D) did not all overlap with those from the *in vitro* assay. This might be explained by the subsequent exonucleolytic degradation of mRNA that occurs only *in vivo* or by slightly suboptimal conditions chosen for the *in vitro* assay.

### Programming the system for targeted transcriptional silencing

To investigate if CRISPR-mediated RNA interference can also be programmed to degrade mRNA of specific chromosomal genes, artificial miniCRISPR loci with and without a spacer targeting the mRNA of β-galactosidase (β-gal) were constructed and expressed in *S. solfataricus*. The CRISPR leader region, the repeats and the spacers stemmed from locus D (44) of the same organism. An additional β-gal-specific spacer of 39 nt was designed such that it hybridized to a region in the mRNA of β-gal whose eight adjacent nucleotides exhibited complementarity to the repeat-derived 5′-handle of the crRNA (construct miniCRISPR-BG-HA, Figure 2A and D). This construct thus followed the same principle to suppress DNA interference as used in D63-HA earlier. It exhibited only two mismatches within the 8-nt sequence, which was still sufficient to fully suppress DNA interference, as shown in (33). On transfection of *S. solfataricus* with the recombinant virus and a control virus with the same miniCRISPR locus but lacking spacer BG-HA, cells were split and grown in two different sugar-containing media that yielded different amounts of β-gal mRNA in the cells. Figure 2B displays the amount of β-gal mRNA compared with that of the control, as estimated by qPCR. The cells were grown in tryptone media and rich media (tryptone/arabinose) that induce slightly different absolute mRNA levels in the culture (∼3-fold higher with arabinose). Depending on the growth medium, 52% (tryptone media) and 41% (tryptone/arabinose media) of β-gal mRNA was found for BG-HA compared with the control cells. In line with this finding, between 45 and 49% of relative protein activity was detected in crude extracts of the same cell preparations, demonstrating that the gene was effectively silenced under both growth conditions. Similar levels of mRNA cleavage as seen here have been obtained earlier in eukaryotes. For example, between 50 and 75% of the mRNA level was decreased in human breast cancer cell lines by using a variety of small RNAs, endogenous micro RNA and small interfering RNA or exogenous micro RNA mimics and duplex RNA (45). Again we demonstrated a crRNA-dependent cutting of the mRNA at UA and UU sites by the CMR complex *in vitro* (Figure 2D and Supplementary Figure S2B).

## CONCLUSION

Our experiments confirm CRISPR-mediated RNA-cleavage as suggested earlier through *in vitro* results with purified CMR complexes in two different archaea (28,30). We demonstrate here in addition that the RNA interference can be programmed *in vivo* to target mRNA degradation of a specific chromosomal gene. Thus, CRISPR-mediated RNA interference can be used in this archaeon and probably many other bacteria and archaea to downregulate chromosomal genes on the post-transcriptional level via a CMR complex. Our data point to the possibility that the CRISPR system might generally interfere with gene expression in its host. It also implicates interesting applications in future research to investigate the function of essential genes in archaea and bacteria, provided that DNA targeting can be abolished. We assume that the use of several spacers targeted to the same gene will increase the silencing effect to higher levels than observed here. For this purpose, the use of a *Sulfolobus* strain solely expressing a CMR-like CRISPR type would be most suitable. Our system as well as the recently demonstrated programmable repression systems in bacteria (26,27) might become interesting additions to the genetic toolboxes of bacteria and archaea in the future. Furthermore, detailed studies of the underlying mechanism of RNA interference *in vivo* and of the effects of RNA interference on virus propagation are now becoming possible.

## SUPPLEMENTARY DATA

Supplementary Data are available at NAR Online.

## FUNDING

European SulfoSYS-project [SysMo P–N-01-09-23] and grant [9P23000 and P25369] by the Austrian Research fund (to C.S.) and by grant [BB/K000314/1] from the Biotechnology and Biological Sciences Research Council (to M.F.W.). Funding for open access charge: Austrian Science Fund.

*Conflict of interest statement*. None declared.

## Supplementary Material

Supplementary Data
