# Supplementary material for: CRISPR-mediated targeted mRNA degradation in the archaeon Sulfolobus solfataricus
Source: Nucleic Acids Res. 2014 Mar 6;42(8):5280–8. doi: 10.1093/nar/gku161 (PMC4005642; doi:10.1093/nar/gku161)
Supplement: Supplementary Data [file supp_42_8_5280__index.html]

CRISPR-mediated targeted mRNA degradation in the archaeon Sulfolobus solfataricus — CRISPR-mediated targeted mRNA degradation in the archaeon Sulfolobus solfataricus — Supplementary Data 

# CRISPR-mediated targeted mRNA degradation in the archaeon *Sulfolobus solfataricus*

## Supplementary Data

files

**Files in this Data Supplement:**

- Supplementary Data - docx file
