# Supplementary material for: CRISPR-mediated targeted mRNA degradation in the archaeon Sulfolobus solfataricus
Source: Nucleic Acids Res. 2014 Mar 6;42(8):5280–8. doi: 10.1093/nar/gku161 (PMC4005642; doi:10.1093/nar/gku161)
Supplement: Supplementary Data [file supp_gku161_nar-03498-h-2013-File004.docx]

**CRISPR-mediated targeted mRNA degradation *in vivo***

**Supplementary information:**

Figure S1, S2, S3, S4, S5

Table S1, S2, S3

**A**


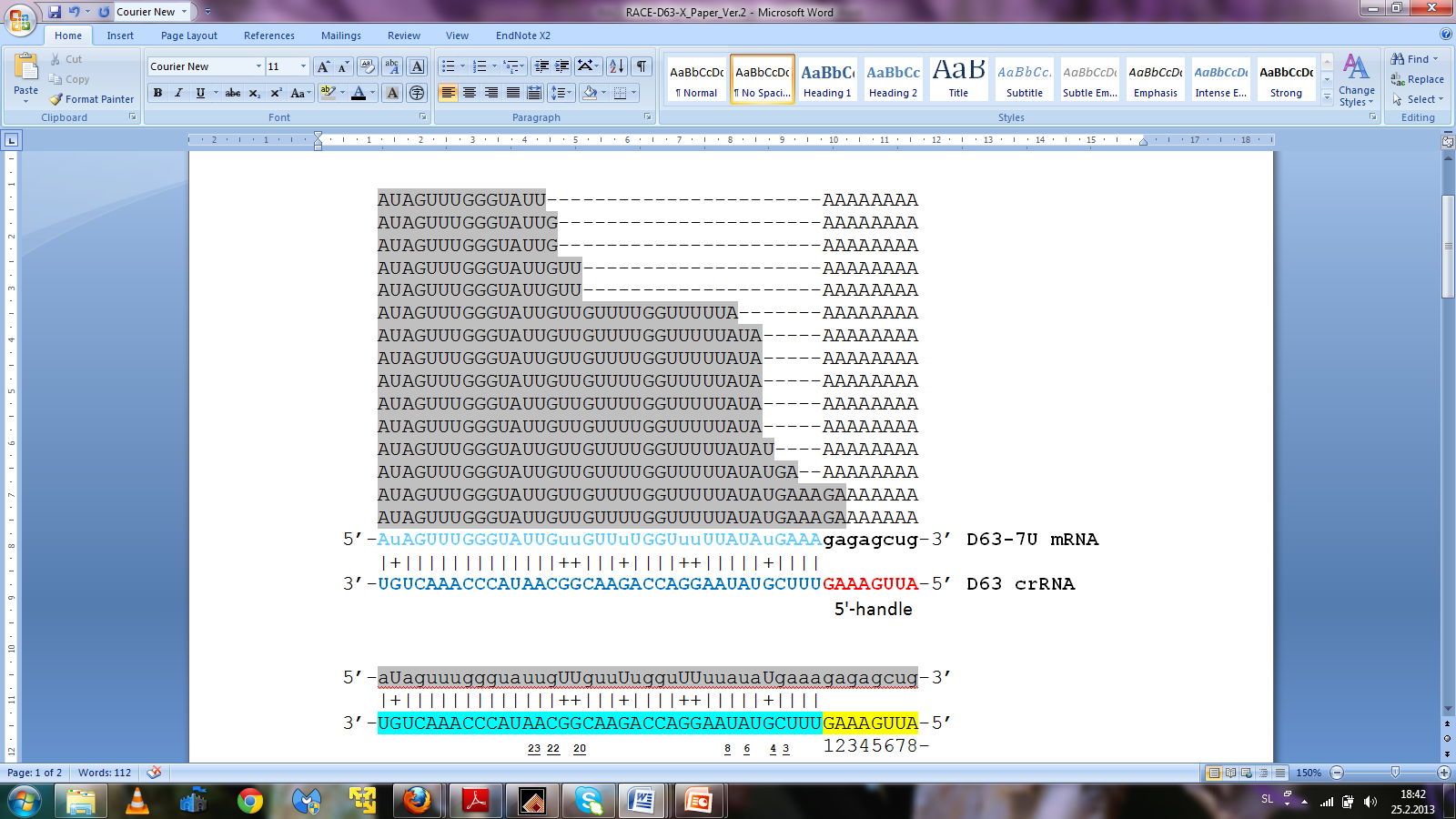


**B**


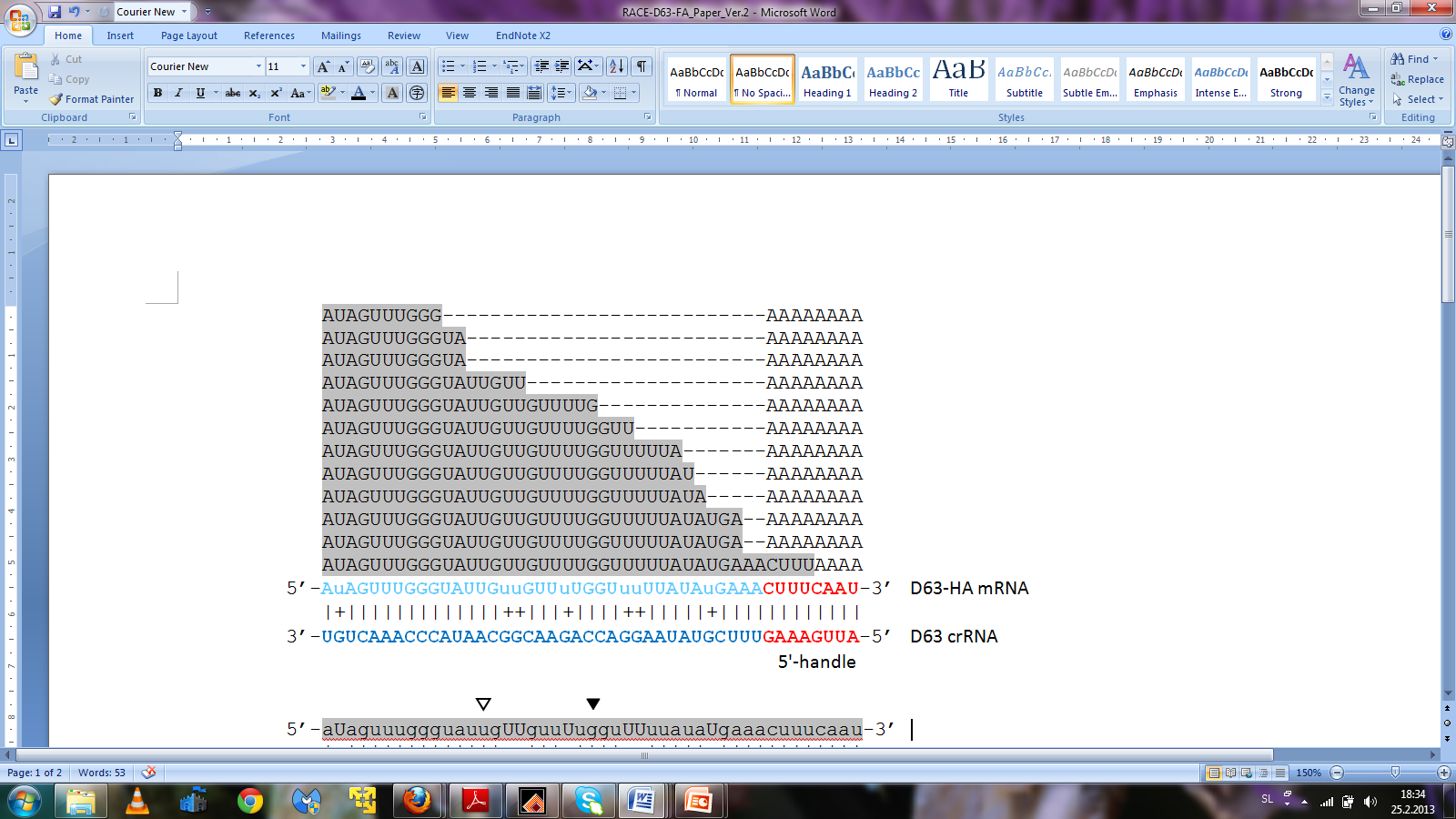


**Figure S1.** Result of RACE experiment (rapid amplification of cDNA ends) showing the mapped 3’ends of mRNA (grey shading) from transfectants of construct D63-7U (A) and D63-HA (B) that were located inside or closely flanking the protospacer sequence (for more details see suppl. material). The protospacer region (light blue) and flanking 8nt (black) as well as the respective crRNA are displayed below (dark blue and red, respectively). Mismatches between both RNAs are in lowercase letters, matching nucleotides in capitals.

**A** **B**


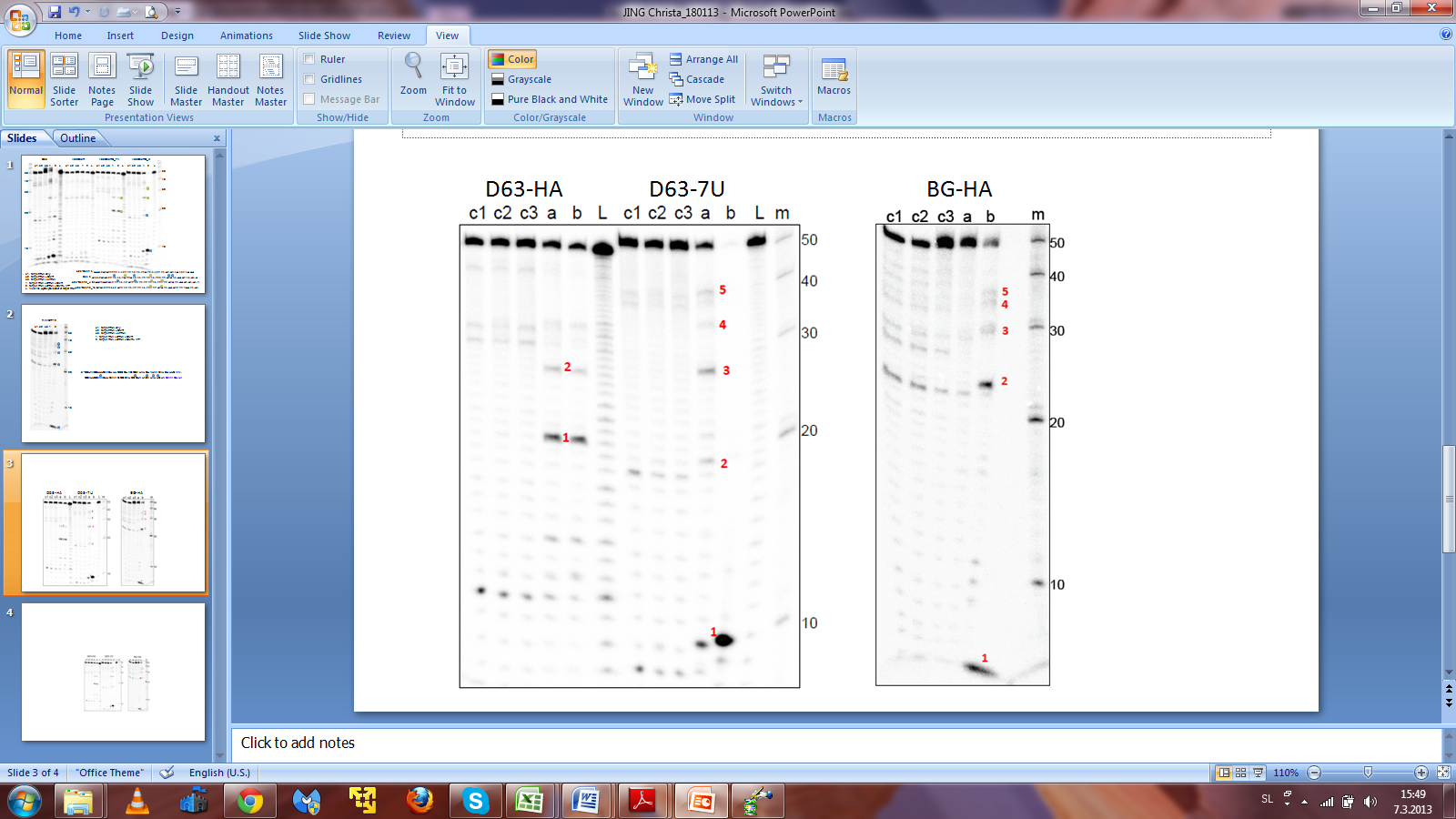

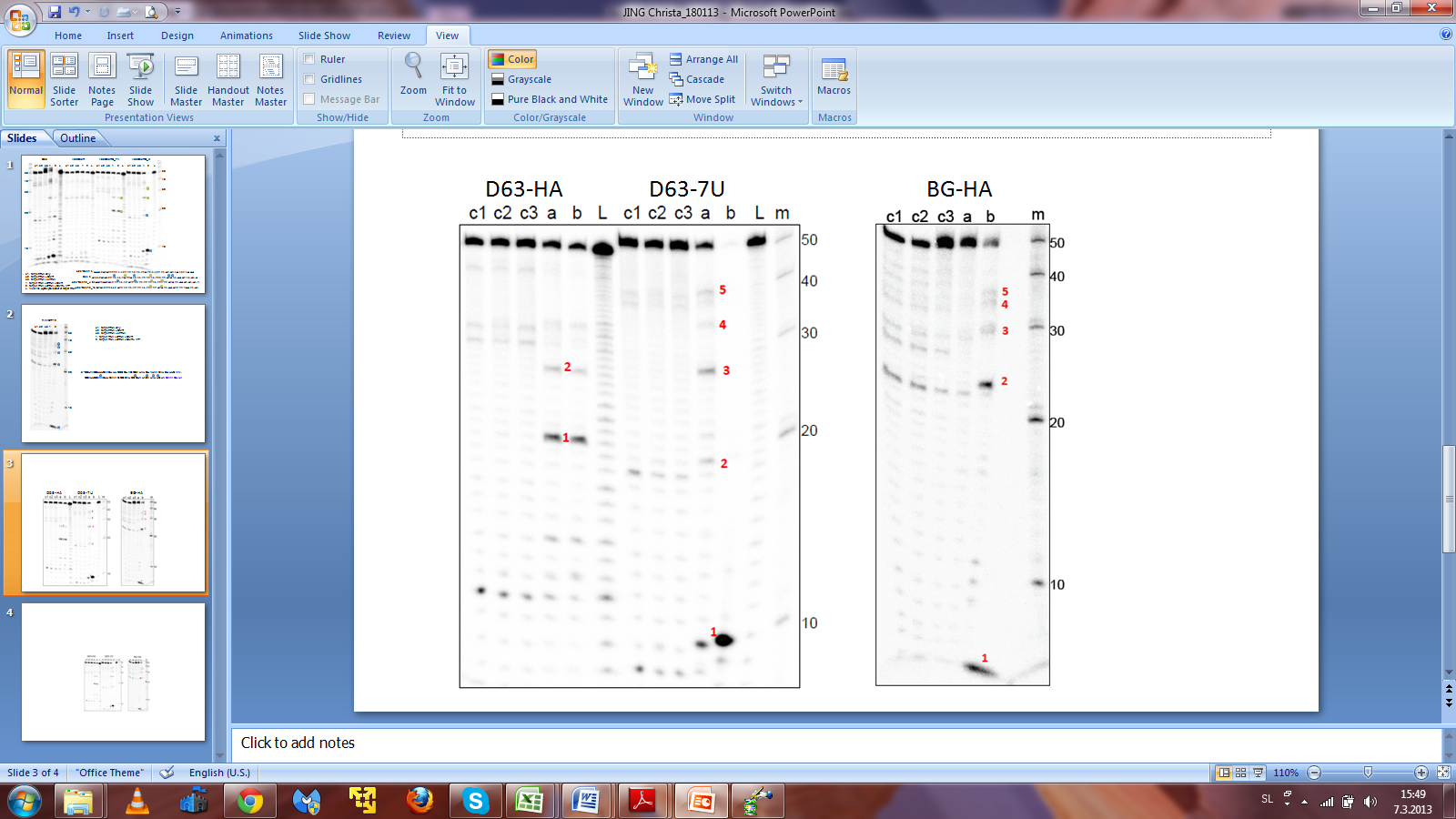


**Figure S2.** In vitro cleavage by CMR Phosphorimages showing the in vitro cleavage of D63-HA and, D63-7U (A) and of miniCRISPR-BG-HA (B) by the purified CMR complex. Cleavage sites (red numbers) were mapped to the substrates and are shown in Fig. 1D and 2D of the main paper. Lanes are: c1, target RNA alone; c2, target RNA with CMR complex; c3, target RNA with crRNA; a, assay in the absence of ATP; b, assay in the presence of ATP; m, RNA size markers; L, target RNA hydrolytic ladder. The cleavage activity of D63-7U and BG-HA was strongly stimulated by ATP, while the cleavage of D63-HA was not (presumably due to the effect of complete base pairing between the 5’-handle of crRNA and its target in the latter construct). These data suggest that the CMR complex does not have an absolute requirement for a mismatch opposite the 5’-handle. This is not that surprising as there is no aboslute requirement to discriminate self from non-self when RNA is targeted.


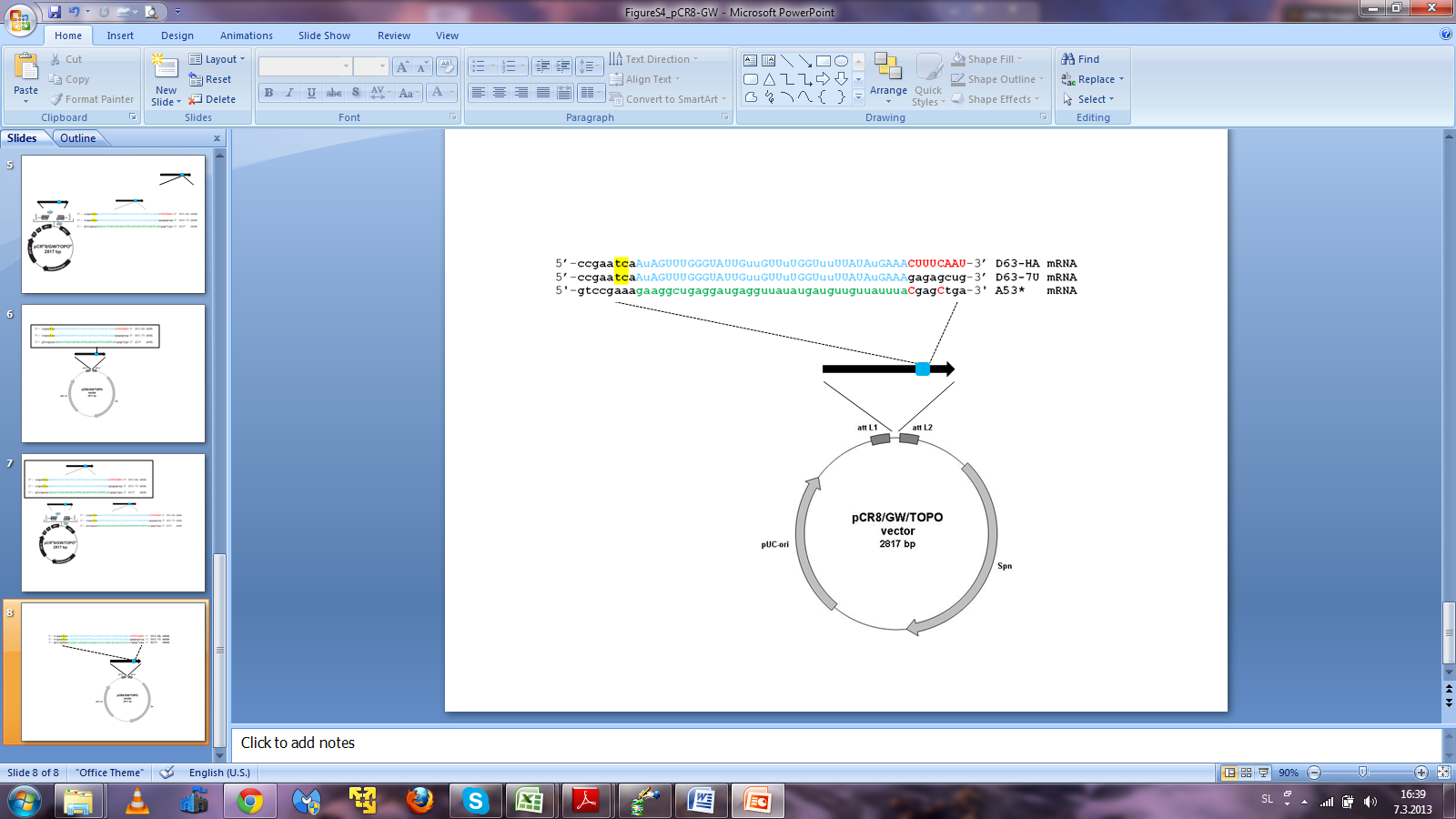


**Figure S3.** Gateway entry vector (Invitrogen™) with inserts of the three different constructs D63-HA, D63-7U and A53* displayed above. Color code as in main manuscript, i.e. blue: protospacer with matching crRNA in strain P1, green protospacer without match, red: 8nt handle, capitals: match between crRNA and target (=protospacer), lowercase letter: mismatch.

**A B**


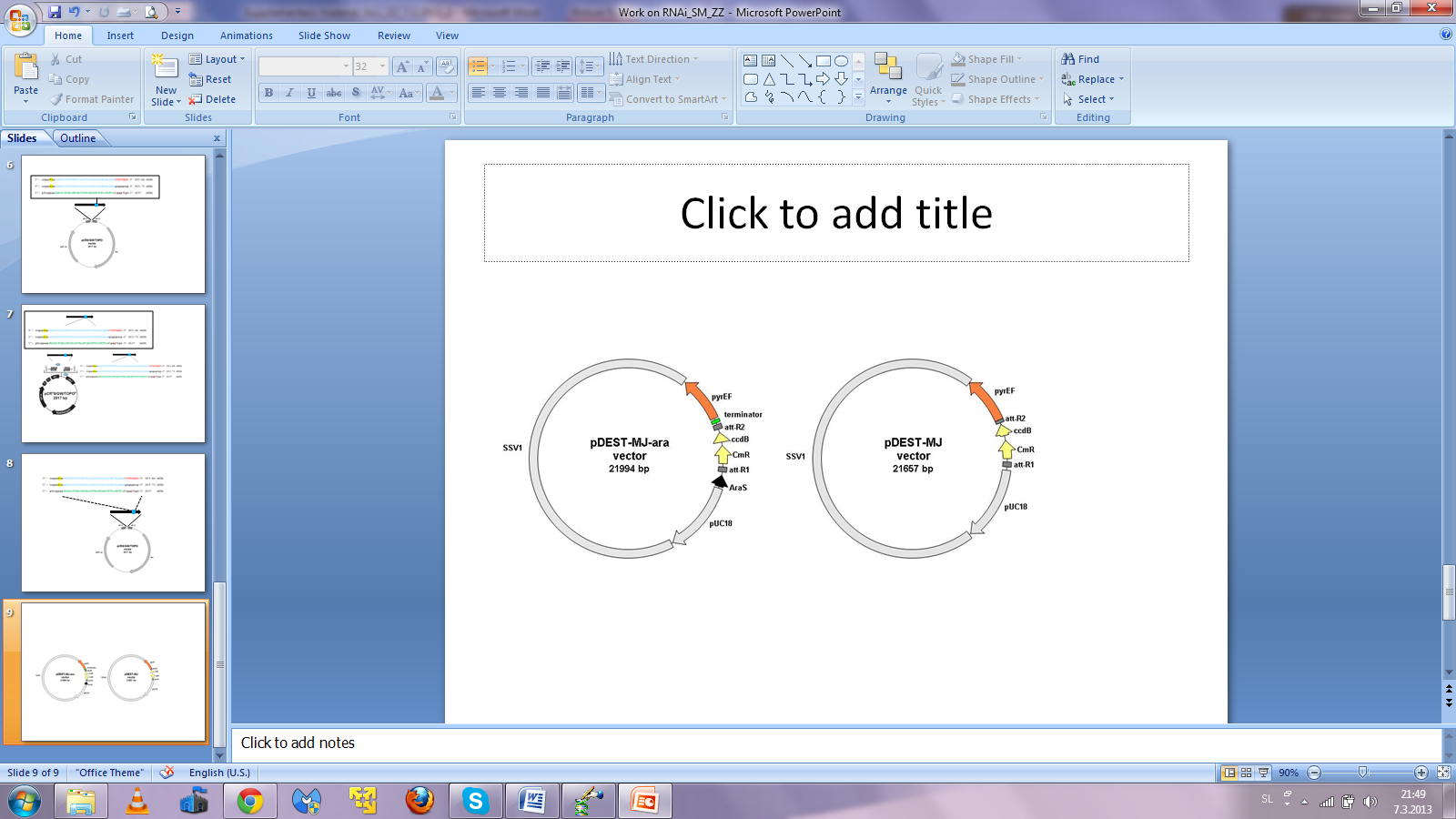


**Figure S4.** Gateway destination vectors, i.e. shuttle vectors between E. coli and S. solfataricus based on the Sulfolobus SSV1 virus and pUC18. (A) pDEST-MJ-ara used for constructs D63-HA/7U and A53*, (B) pDEST-MJ used for miniCRISPR control and BG-HA. For more information see suppl. materials and methods.

**Figure S5.** Total RNA separation on a denaturing-Agarose gel, before transferred to a Nylon membrane in order to perform the Northern-blot analysis (Figure 1B).

**Table S1. Total amount of protospacer mRNA (A) and DNA (B) in cells transfected with constructs D63-7U, D63-HA or A53*, respectively* as estimated by qPCR (see materials and methods).**

**A B**

| Construct | Average per µg RNA (Q2) | s.d. |
| --- | --- | --- |
| D63-7U | 5,77E+05 | 2,60E+05 |
| D63-HA | 1,05E+06 | 3,52E+05 |
| A53* | 2,67E+06 | 1,30E+06 |

| Construct | Average per µg DNA (Q1) | s.d. |
| --- | --- | --- |
| D63-7U | 3,07E+06 | 7,82E+05 |
| D63-HA | 1,25E+07 | 2,17E+06 |
| A53* | 7,81E+06 | 2,21E+06 |

*n*=3 biological replicates, with each *n*=3 technical replicates, values given per total RNA and DNA, respectively, s.d.= standard deviation.

**Table S2. Information on PCR (polymerase chain reactions) used in this study.**

| **Primer name** | **Application** | **Product  Size** | **Template** | **Construct** |
| --- | --- | --- | --- | --- |
| Q1_FW | Q-PCR | 283 | ORF406 | / |
| Q1_RW | Q-PCR | 283 | ORF406 | / |
| Q2_FW | Q-PCR | 200 | ORF406 | / |
| Q2_RW | Q-PCR | 200 | ORF406 | / |
| BG-Q1_FW | Q-PCR | 305 | B-gal | / |
| BG-Q1_RW | Q-PCR | 305 | B-gal | / |
| BG-Q2_FW | Q-PCR | 267 | B-gal | / |
| BG-Q2_RW | Q-PCR | 267 | B-gal | / |
| 406-OE-FW | OE | / | ORF406 | D63-7U & D63-HA |
| 406-OE-Rw | OE | / | ORF406 | D63-7U & D63-HA |
| D63-7U_FW | OE | / | ORF406 | D63-7U |
| D63-7U_RW | OE | / | ORF406 | D63-7U |
| D63-HA-Fw | OE | / | ORF406 | D63-HA |
| CR6-FW | PCR | 868 | Chromosome | miniCR |
| CR6-RW | PCR | 868 | Chromosome | miniCR |
| M_Fw | OE | / | miniCR | miniCR-BG-HA |
| M_Rw | OE | / | miniCR | miniCR-BG-HA |
| MOE_Fw | OE | / | miniCR | miniCR-BG-HA |
| MOE_Rw | OE | / | miniCR | miniCR-BG-HA |
| BG-HA_Fw | OE | / | miniCR | miniCR-BG-HA |
| BG-HA_Rw | OE | / | miniCR | miniCR-BG-HA |
| Q1_FW | RACE | ND | ORF406 | / |
| 406_FW3 | RACE | ND | ORF406 | / |
| dT_AnP_RW | RACE | ND | ORF406 | / |
| Pro-FW | Northern-blot | 333 | ORF406 | / |
| Pro-RW | Northern-blot | 333 | ORF406 | / |

ND = not defined.

**Table S3. Sequences of primers used in this study.**

| **Primer name** | **Sequence 5'-3'** |
| --- | --- |
| Q1_FW | GGTTACATAAGGCTCTGTCGAGG |
| Q1_RW | TTGCCGTACTTCTCAAGCTGG |
| Q2_FW | CACTCTACGCTGGGCAGACATCT |
| Q2_RW | TTGGGAACCTGGCTAGACCTTCA |
| BG-Q1_FW | TTTACTGGACCAAGTGGTTGGCT |
| BG-Q1_RW | GGCTGGAATGAGCTATTAGCGT |
| BG-Q2_FW | TCAGAAGATCCAAATACTGACTGGT |
| BG-Q2_RW | TCGTTTTCGTTTATCTCAACCTCTGTCAC |
| 406-OE-FW | GTAGTAATGGAGGAGATAGACGTTAAACAGTTGGTGGAAAAGTCCGAAA |
| 406-OE-Rw | GCCGTACTTCTCAAGCTGGTACTTCAAGATGCTTTTCAGCTC |
| D63-7U_FW | AAAACCAAAACAACAATACCCAAACTATTGATTCGGACTTTTCCACCAAC |
| D63-7U_RW | GGTATTGTTGTTTTGGTTTTTATATGAAAGAGAGCTGAAAAGCATCTTGA |
| D63-HA-Fw | TTGTTGTTTTGGTTTTTATATGAAACTTTCAATGAGAGCTGAAAAGCATC |
| CR6-FW | TTATCGGAGGCATATAATAGTTCCA |
| CR6-RW | AATCCAATGAGCCGGGACAAGTTTCACAA |
| M_Fw | TGCAGAATTATCGCCCAGAACAA |
| M_Rw | GTTAGTTCACCCACCGACAAATACA |
| MOE_Fw | AGAATTATCGCCCAGAACAAATTTCTGATAATCTCTTATAGAATTGAAAG |
| MOE_Rw | GTTAGTTCACCCACCGACAAATACAACTTTCAATTCTATAAGAGATTATC |
| BG-HA_Fw | TATGCCTACGGGAAAGTTCAAAGCTTAGGGATAATCTCTTATAGAATTGA |
| BG-HA_Rw | GAACTTTCCCGTAGGCATATGTATAACATCTTTCAATTCTATAAGAGATT |
| 406_FW3 | AAGGTCTAGCCAGGTTCCCA |
| dT_AnP_RW | GACTCGTGGAGCATGACCTCGTTTTTTTTTTTTTTTTTT |
| Pro-FW | AATTCCCACAATTGCCAAAG |
| Pro-RW | TTTCGGACTTTTCCACCAACT |
